# Disseminated Histoplasmosis, Pulmonary Tuberculosis, and Cytomegalovirus Disease in a Renal Transplant Recipient after Infection with SARS-CoV-2

**DOI:** 10.1155/2022/8042168

**Published:** 2022-08-28

**Authors:** Carvallo-Venegas Mauricio, Fuentes-López Elsa Angélica, Andrade-Ortega Antonio de Jesús, Torres-Baranda José Rodrigo, Carrasco-Carrizosa Aldo, Cerrillos-Gutierrez José Ignacio, Andrade-Sierra Jorge

**Affiliations:** ^1^Department of Nephrology and Organ Transplant Unit, Specialties Hospital, National Western Medical Centre, Mexican Institute of Social Security, Mexico; ^2^Department of Microbiology, University Health Sciences Center, University of Guadalajara, Guadalajara, Jalisco, Mexico; ^3^Department of Respiratory Diseases, “Valentin Gómez Farias” Hospital, ISSSTE, Guadalajara, Jalisco, Mexico; ^4^Department of Physiology, University Health Sciences Center, University of Guadalajara, Guadalajara, Jalisco, Mexico

## Abstract

**Introduction:**

Infection with SARS-CoV-2 increases the risk of acute graft dysfunction (AGD) in renal transplant recipients (RTR), and the risk of concurrently presenting with opportunistic infections is also increased. There is no current consensus on the management of immunosuppression during SARS-CoV-2 infection in RTR. *Case Presentation*. A 35-year-old male RTR from a living related donor presented with SARS-CoV-2 infection (January 2021). Two months later, despite alterations to his immunosuppression regimen (tacrolimus (TAC) was reduced by 50%, and the mycophenolic acid (MMF) was suspended with the remission of symptoms), the patient presented with pulmonary tuberculosis, pneumonia due to respiratory syncytial virus (RSV), cytomegalovirus (CMV) pneumonitis, and histoplasmosis (HP). Management was initiated with antituberculosis medications, ganciclovir, antibiotics, and liposomal amphotericin B, and the immunosuppressants were suspended, yet the patient's evolution was catastrophic and the outcome fatal.

**Conclusion:**

We recommend that in RTR post-COVID-19, the immunosuppression regimen should be gradually reinstated along with strict vigilance in observing for highly prevalent coinfections (TB, HP, and CMV).

## 1. Introduction

From March 2020 to the present date, infection with the coronavirus that causes severe acute respiratory syndrome (SARS-CoV-2) has been a worldwide public health problem associated with multiple complications [1]. In renal transplant recipients (RTR), infection with SARS-CoV-2 increases the risk of acute graft dysfunction (AGD) and higher mortality [2]. In addition to complications from the same SARS-CoV-2 infection, the risk of concurrently presenting with opportunistic infections is also increased. A meta-analysis [3] reported the prevalence of concurrent infections as follows: viral (10%), bacterial (8%), and fungal (4%). On the other hand, the prevalence of delayed infections was primarily bacterial (20%), fungal (8%), and viral (4%), with reports of *Acinetobacter* spp. (22.0%), *Pseudomonas* (10.8%), *Escherichia coli* (6.9%); rhinovirus; and infections with *Candida* spp., as the most prevalent. Similarly, superinfections of *Mycobacterium tuberculosis* [4] and *Histoplasma capsulatum* [5] have been described and associated with high morbidity-mortality in RTR [6, 7]. The risk factors associated with superinfection in SARS-CoV-2 include previous pulmonary illness, encephalopathy, mechanical ventilation, pharmacological immunosuppressants, and prolonged hospital stay [8]. There is no current consensus on the management of immunosuppression during SARS-CoV-2 infection. Some recommendations in cases of moderate illness include suspension of the antimetabolite and continuing with low doses of calcineurin inhibitors (CNI), reserving the suspension of both for serious cases [9, 10]. It is recommended that the use of corticosteroids (prednisone) is changed to dexamethasone in cases that require supplementary oxygen therapy [11]. Despite these measures, the risk of superinfection is high, and RTR patients require strict vigilance.

The case presented corresponds to a male first-time RTR from a living related donor, who presented with SARS-CoV-2 infection and, 2 months later, presented with pulmonary tuberculosis and pneumonia due to respiratory syncytial virus (RSV), cytomegalovirus (CMV) pneumonitis, and histoplasmosis (HP).

## 2. Case Presentation

A 35-year-old male RTR (in March 2019) with high serological risk for CMV (donor IgG+/recipient IgG-) received induction immunosuppression based in basiliximab (BSL) and maintenance with tacrolimus (TAC) 0.10 mg/kg/day, mycophenolic acid (MMF) 1 g every 12 hours, and prednisone (PDN) 5 mg every 24 hours. The patient's prophylaxis treatment with valganciclovir (VGC) 900 mg/day for 6 months and the evolution of the transplant were satisfactory, with an average creatinine of 1.2 mg/dl and without events of acute rejection (AR). In January 2021, he presented with SARS-CoV-2 (RT-PCR +) and fever for 3 days (37.7-38°C), without oximetry alterations and with the following management: paracetamol *per os* (PO) in case of fever, ivermectin 6 mg PO every 12 hours for 3 days, azithromycin 500 mg PO every 24 hours for 3 days, melatonin 5 mg PO every 24 hours, zinc 50 mg PO every 12 hours, vitamin D 5000 IU PO every 24 hours, and famotidine 20 mg PO every 12 hours. In addition, the TAC dose was reduced by 50%, and the antimetabolite (MMF) was suspended. Prednisone was maintained at 5 mg per day, with the remission of symptoms.

**Figure 1:**
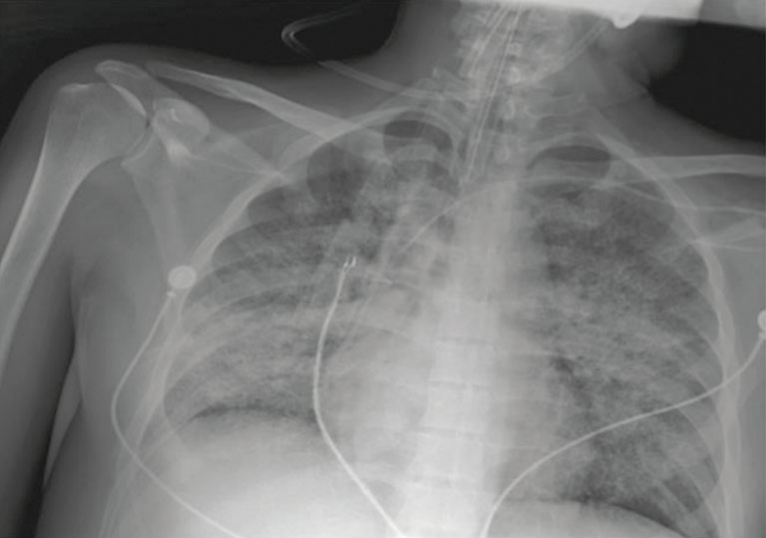
Chest X-ray with an extensive nodular pattern in both lung fields.

**Figure 2:**
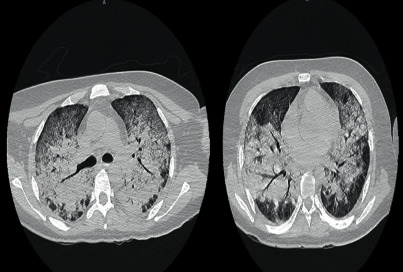
Chest tomography with generalized infiltrate and centripetal distribution to the bronchial tree with a non-micronodular pattern.

In April 2021, the patient's fever returned (39°C), predominantly at night, and he experienced shivers, diaphoresis, diarrhea, unwellness, general hyporexia, dry cough, dyspnea, and weight loss, requiring hospitalization and initiation of the diagnostic work-up with RT-PCR for SARS-CoV-2 with negative results. A Chest X-ray (Figure 1) showed an extensive nodular pattern in both lung fields, and chest tomography (Figure 2) showed a predominantly central pattern of bilateral ground glass opacities.

Bronchoscopy with bronchoalveolar lavage was performed with the PCR result positive for *Mycobacterium tuberculosis* (*MTb*), RSV, and CMV. Management with antituberculosis medications was initiated (rifampin 600 mg, isoniazid 300 mg, pyrazinamide 1600 mg, and ethambutol 1200 mg every 24 hours; ganciclovir 5 mg/kg IV every 12 hours; and antibiotics adjusted to renal function (meropenem 1 g IV every 12 hours, levofloxacin 500 mg IV every 48 hours, and linezolid 600 mg IV every 12 hours)), and the immunosuppressants were suspended (TAC and MMF), continuing only with PDN 5 mg per day. The patient's evolution was unfavorable with progressive dyspnea, persistent fever, tachycardia (140 beats/minute), hypoaeration in both pulmonary fields, and oxygen saturation of 80% despite the use of high-flow nasal cannulas (FiO_2_ 100%), as well as the need for mechanical ventilation (MVI) and the use of norepinephrine (average dose of 0.1 mcg/kg/min) for septic shock. The patient's biochemical characteristics on hospitalization and during evolution are shown in Table 1.

**Figure 3:**
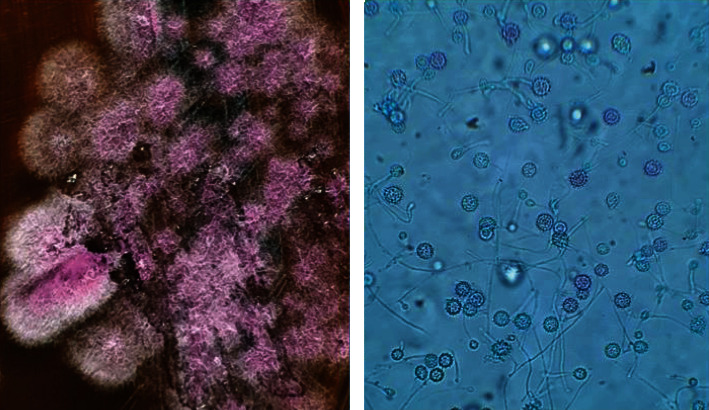
Macroscopic and microscopic views of *H. capsulatum* from the bronchoalveolar lavage.

Seven days later, due to the lack of clinical response, empirical treatment with liposomal amphotericin B was initiated (0.14 mg/kg/d) with clinical improvements in the ventilatory parameters. The final report provided 10 days after the initiation of therapy with amphotericin B showed the growth of *Histoplasma capsulatum* in the cultivation medium of the bronchoalveolar lavage done 4 days after hospitalization (Figure 3).

However, the final evolution was deterioration of the respiratory pattern due to bacterial superinfection (*Acinetobacter baumannii* and *Klebsiella pneumoniae*) isolated through bronchoalveolar lavage obtained 5 days after MVI, with a multi-drug-resistant profile and sensitivity only to polymyxins and tigecycline at a minimum inhibitory concentration (MIC; *μ*g/ml) < 2 and ≤1, respectively. Therefore, the decision made by our medical team was to initiate colistin (polymyxin E) at a loading dose of 3.5 mg/kg/d and a maintenance dose of 2.5 mg/k/d, divided into two doses, and tigecycline at an initial dose of 100 mg and a maintenance of 50 mg every 12 hours. Nevertheless, the patient evolution was catastrophic, and the outcome was fatal.

## 3. Discussion

Tuberculosis (TB) and histoplasmosis (HP) are frequent infections in solid organ transplant patients, with an incidence of 5 per 1000 person-years for TB [6] and an incidence of 1 per 1000 person-years for HP [12]. The risk for these types of infections is higher in the first posttransplant years and is associated with immunosuppression based on TAC and MMF [13]. Coinfection with both pathogens is described in 8-15% of patients with human immunodeficiency virus (HIV) [14], but there are no reports on the prevalence of this coinfection in patients who are recipients of solid organ transplants. The present case corresponds to a concurrent infection with TB and HP, having, as a primary risk factor, exposure to immunosuppressants and a previous infection with SARS-CoV-2. In RTR, infection with SARS-CoV-2 is associated with a mortality of 32%, requirements for MVI, and a high percentage of acute renal injury (ARI) [2]. The present case had the history of a mild infection with SARS-CoV-2. We consider that this may have favored the appearance of simultaneous coinfections despite the suspension and/or reduction of the immunosuppressants that were managed according to the recommendations of some experts who suggest a reduction of the immunosuppressant doses in mild illness or suspension of one or two immunosuppressants in moderate-severe illness and subsequent reinstatement scaled according to clinical improvement [9, 10]. In RTR, mortality varies between 9.5% and 40% with TB [15], 10% with HP [7], and 10% with CMV [16]. In the present case, mortality is greater due to multiple opportunistic pathogens in addition to the nosocomial multi-drug-resistant pathogens that contributed to the fatal outcome. The SARS-CoV-2 infection favors a state of immunosuppression [17] and is associated with secondary infections including active TB and active HP [3–5]. The mechanisms involved are cellular lesions in the respiratory airways provoked by SARS-CoV-2 (apoptosis, destruction of the integrity of the epithelium, and disturbance of the polarity of the cilia), leading to deficient mucociliary clearance with a subsequent augmentation in risk of secondary infections [18]. Similarly, the virus has an affinity for lymphocytes, inducing apoptosis and lymphocytopenia in the host. Other mechanisms by which the virus is associated with the deterioration of the immunological system are the production of tumor necrosis factor alpha and interleukins 2 and 6, which induce apoptosis of the lymphocytes, and lastly, the virus has been associated with myelopoiesis, favoring the production of immature and dysfunctional myeloid cells that inhibit the proliferation of T lymphocytes [19]. These aforementioned mechanisms conditioned the present case to have coinfections, since the protective immunity against TB is dependent on T CD4+ lymphocytes [20]. Our team did not measure subpopulations of lymphocytes, but the patient, during active infection, presented with lymphocytopenia throughout hospitalization. On the other hand, HP infection is associated with states of immunosuppression and subjacent pulmonary disturbances, which are conditions that are present due to the aforementioned pathophysiological processes induced by SARS-CoV-2. Risk factors for the multiple infectious picture demonstrated by this case included exposure to immunosuppressants necessary for the transplant and the history of a recent SARS-CoV-2 infection.

In a scenario like this, the search for latent TB after an infection with SARS-CoV-2 should be made in those recently exposed to an active TB case, personnel at microbiology laboratories, residents of countries with known high TB rates, and/or high-risk patients [21]. We consider that SARS-CoV-2 infection is an additional risk factor; therefore, performing a test for latent TB and treating positive cases after discarding active TB are recommended. Lastly, a strategy for prevention of CMV infection after SARS-CoV-2 is not yet described beyond the immediate posttransplant recommendations (prophylactic therapy or a preemptive strategy in the first 3-6 months) according to serologic risk or after antibody therapy with antilymphocyte antibodies [22].

## 4. Conclusions

In renal transplant recipients post-COVID-19, we recommend reinstating immunosuppression gradually as well as strict vigilance in observing for highly prevalent coinfections (TB, HP, and CMV) in endemic areas based on transplant center guidelines. We consider that the added risk due to infection with SARS-CoV-2, and the potential use of prophylaxis in this population, requires further scientific evidence.

## Figures and Tables

**Table 1:** Biochemical characteristics of the patient.

Laboratory test	Reference range	Hospitalization 05.04.21	Intubation 14.04.21	21.04.21	25.04.21
Hemoglobin	12-17 g/dl	8.7	9.4	9.1	7.5
Platelets	130-400 thousands/*μ*l	69	145	127	81
Leukocytes	4-11 thousands/*μ*l	4.66	19.94	17.17	17.19
Neutrophils	2-10 thousands/*μ*l	3.96	17.57	14.9	5.27
Lymphocytes	1-4 thousands/*μ*l	0.36	0.86	0.97	0.81
Procalcitonin	<0.05 ng/ml	1	19.59	35.9	35.78
Urea	20-43 mg/dl	23.98	47.96	107.9	83.9
Creatinine	0.65-1.24 mg/dl	0.87	1.48	2.9	2.46
K	3.6-5 mmol/l	4.3	4.4	4.8	4.3
Na	137-145 mmol/l	129	131	137	141

## Data Availability

The manuscript did not contain any individual, personally identifiable data in any form. All data generated or analyzed during this study are included in this case report.
